# Effect of a low versus intermediate tidal volume strategy on pulmonary complications in patients at risk of acute respiratory distress syndrome—a randomized clinical trial

**DOI:** 10.3389/fmed.2023.1172434

**Published:** 2023-06-07

**Authors:** Candelaria de Haro, Ary Serpa Neto, Gemma Gomà, Maria Elena González, Alfonso Ortega, Catalina Forteza, Fernando Frutos-Vivar, Raquel García, Fabienne D. Simonis, Federico Gordo-Vidal, David Suarez, Marcus J. Schultz, Antonio Artigas

**Affiliations:** ^1^Intensive Care Department, Hospital Universitari Parc Taulí, Institut d’Investigació i Innovació Parc Taulí (I3PT-CERCA), Universitat Autònoma de Barcelona, Sabadell, Spain; ^2^CIBER Enfermedades Respiratorias, Instituto de Salud Carlos III, Madrid, Spain; ^3^Department of Intensive Care, Amsterdam University Medical Centers, Location ‘AMC’, Amsterdam, Netherlands; ^4^Department of Critical Care Medicine, Hospital Israelita Albert Einstein, São Paulo, Brazil; ^5^Department of Critical Care Medicine, Australian and New Zealand Intensive Care Research Centre (ANZIC-RC), Monash University, Melbourne, VIC, Australia; ^6^Department of Critical Care, Data Analytics Research and Evaluation (DARE) Centre, Austin Hospital, Melbourne, VIC, Australia; ^7^Intensive Care Unit, Hospital Universitario de Torrejón, Madrid, Spain; ^8^Intensive Care Unit, Hospital Universitario Puerta de Hierro, Majadahonda, Spain; ^9^Intensive Care Unit, Hospital Son Llàtzer, Palma de Mallorca, Spain; ^10^Intensive Care Unit, Hospital Universitario de Getafe, Getafe, Spain; ^11^Reanimation Unit, Hospital Universitario 12 de Octubre, Madrid, Spain; ^12^Intensive Care Unit, Hospital del Henares, Grupo de Investigación en Patología Crítica de la Universidad Francisco de Vitoria, Pozuelo de Alarcón, Madrid, Spain; ^13^Department of Medical Affairs, Hamilton Medical AG, Bonaduz, Switzerland; ^14^Mahidol-Oxford Tropical Medicine Research Unit (MORU), Mahidol University, Bangkok, Thailand; ^15^Nuffield Department of Medicine, University of Oxford, Oxford, United Kingdom

**Keywords:** intensive care, critical care, mechanical ventilation, lung protection, tidal volume, ARDS, mortality

## Abstract

**Introduction:**

There is no consensus on whether invasive ventilation should use low tidal volumes (V_T_) to prevent lung complications in patients at risk of acute respiratory distress syndrome (ARDS). The purpose of this study is to determine if a low V_T_ strategy is more effective than an intermediate V_T_ strategy in preventing pulmonary complications.

**Methods:**

A randomized clinical trial was conducted in invasively ventilated patients with a lung injury prediction score (LIPS) of >4 performed in the intensive care units of 10 hospitals in Spain and one in the United States of America (USA) from 3 November 2014 to 30 August 2016. Patients were randomized to invasive ventilation using low V_T_ (≤ 6 mL/kg predicted body weight, PBW) (*N* = 50) or intermediate V_T_ (> 8 mL/kg PBW) (*N* = 48). The primary endpoint was the development of ARDS during the first 7 days after the initiation of invasive ventilation. Secondary endpoints included the development of pneumonia and severe atelectases; the length of intensive care unit (ICU) and hospital stay; and ICU, hospital, 28– and 90–day mortality.

**Results:**

In total, 98 patients [67.3% male], with a median age of 65.5 years [interquartile range 55–73], were enrolled until the study was prematurely stopped because of slow recruitment and loss of equipoise caused by recent study reports. On day 7, five (11.9%) patients in the low V_T_ group and four (9.1%) patients in the intermediate V_T_ group had developed ARDS (risk ratio, 1.16 [95% CI, 0.62–2.17]; *p* = 0.735). The incidence of pneumonia and severe atelectasis was also not different between the two groups. The use of a low V_T_ strategy did neither affect the length of ICU and hospital stay nor mortality rates.

**Conclusions:**

In patients at risk for ARDS, a low V_T_ strategy did not result in a lower incidence of ARDS than an intermediate V_T_ strategy.

**Clinical Trial Registration**: ClinicalTrials.gov, identifier NCT02070666.

## 1. Introduction

Invasive mechanical ventilation is a commonly applied support in critically ill patients with acute respiratory failure (1, 2). While often life-saving, it has the potential to induce lung injury, especially when too large tidal volumes (V_T_) are used in patients with existing lung injury, i.e., in patients with acute respiratory distress syndrome (ARDS) (3). There is no consensus on whether ventilation with low V_T_ prevents lung injury in patients at risk of ARDS (4).

One retrospective study in patients without ARDS suggested a 1.3 higher chance for the development of ARDS with every ml increase in V_T_ above 6 mL/kg predicted body weight (PBW) (5). A small and prematurely stopped randomized clinical trial in patients without ARDS showed that ventilation with low V_T_ (6 mL/kg PBW) reduced the risk of developing ARDS in comparison to ventilation with an intermediate V_T_ (10 mL/kg PBW) (6). A well-powered randomized clinical trial in critically ill patients without ARDS did not show the clinical benefit of a ventilation strategy that used low V_T_ (4 to 6 mL/kg PBW) regarding the number of days free from ventilation and alive on day 28 when compared to a ventilation strategy that used intermediate V_T_ (8 to 10 mL/kg PBW) (7). Of note, this latter study enrolled patients at low risk of ARDS.

The Early Preventive ventilation strategy in Acute Lung Injury (EPALI) randomized clinical trial was designed to test the hypothesis that a ventilation strategy using a low V_T_ is superior to a ventilation strategy using an intermediate V_T_ with respect to the development of ARDS in patients at risk of this pulmonary complication.

## 2. Materials and methods

### 2.1. Study design

Early Preventive ventilation strategy in Acute Lung Injury was an international, multicenter, two-arm randomized clinical trial in the intensive care units of 10 hospitals in Spain and one hospital in the USA. The Institutional Review Boards of the participating centers approved the study protocol that is available in the online supplement. Written informed consent was obtained from patients’ representatives before any study-related action was taken. The study was registered at clinicaltrials.gov (study identifier NCT02070666), and the statistical analysis plan was finalized before cleaning and closing the database.

### 2.2. Patients

Consecutive patients were screened and were eligible for participation if they (i) were admitted to the intensive care unit (ICU) of a participating hospital; (ii) were aged ≥18 years; and (iii) had a lung injury prediction score (LIPS) of >4 (8). Patients were excluded if their ventilation before randomization lasted >12 h from intubation, or when the criteria of the Berlin definition for ARDS were met (9). Patients were also excluded if they had previous pneumonectomy or lobectomy, severe cranial trauma with a Glasgow Coma Scale of <9 or known cranial hypertension, severe chronic pulmonary disease, and acute pulmonary embolism. Other exclusion criteria were limitations in care, pregnancy, participation in other interventional trials, and previous randomization in this study.

### 2.3. Randomization

Patients were randomized in a 1:1 ratio through an electronic platform (Soltek Consulting SL, Barcelona, Spain). Due to the characteristics of the intervention, blinding was not possible.

### 2.4. Interventions

Patients assigned to the low V_T_ group started with a V_T_ of 6 mL/kg PBW, after which V_T_ was reduced stepwise with 1 mL/kg PBW every 5 min, to a V_T_ of 4 mL/kg PBW. This strategy was continued for at least 7 days or until tracheal extubation if this happened before day 7. During pressure support ventilation, the pressure support level was set to the lowest possible level to achieve the target V_T_ with a minimum of 5 cm H_2_O. If V_T_ increased >8 mL/kg PBW with the lowest level of pressure support, this was accepted. A respiratory rate of up to 35 per min was accepted. In cases of severe apnea, asynchronies, and uncontrollable acidosis, it was allowed to use higher V_T_. Patients assigned to the intermediate V_T_ group started with a V_T_ of 8 mL/kg of predicted body weight, which could be increased up to 10 mL/kg PBW at the discretion of the treating physician to facilitate good patient–ventilator interaction, adequate respiratory rates, and always ensuring safe plateau pressure limits. This strategy was continued for a total of 7 days, or until a patient fulfilled the current definition of ARDS, at which V_T_ was immediately decreased to 6 mL/kg PBW. If the plateau or maximum airway pressure was ≥25 cm H_2_O, V_T_ was lowered until the plateau or peak airway pressure was <25 cm H_2_O. Ventilator adjustments were verified frequently and were readjusted according to the protocol when necessary.

### 2.5. Standard ventilatory care

Controlled ventilation was preferred over spontaneous ventilation in order to assure the target V_T_; however, it was allowed to switch to a spontaneous mode at the discretion of the attending physician, with the necessary adjustments in order to reach the target V_T_. However, it was not allowed to use more sedation or neuromuscular blocking agents. The fraction of inspired oxygen (FiO_2_) and the positive end-expiration pressure (PEEP) were adjusted at the discretion of the attending physician. Weaning protocols of each participating center were followed to decide the best moment for extubation. More information about the study protocol is detailed in the electronic supplement.

### 2.6. Outcomes

The primary endpoint was the development of ARDS during the first 7 days after randomization. Secondary outcomes included the development of pneumonia or severe atelectasis within the first 7 days of enrollment. Other endpoints included the duration of invasive ventilation, length of stay in the ICU and hospital, and the ICU, hospital, 28–day and 90–day mortality.

### 2.7. Definitions

Newly developed ARDS was defined by the Berlin definition for ARDS (9). A diagnosis of pneumonia required the presence of signs of an infiltrate on the daily routine chest X-ray evaluated by a radiologist, where typical radiographic features that favor pneumonia were patchy areas of consolidation or poorly defined multifocal opacities, without volume loss in the non-dependent areas of the chest. Severe atelectasis requires the presence of increased opacities, loss of diaphragm and heart contours, and an increasing volume loss with mediastinal and pulmonary fissure displacement (10).

### 2.8. Power calculation

We originally planned to include a sample of 400 patients. The power analysis was performed taking into account the incidence of ARDS in patients with a LIPS score higher than 4. The planned sample size of 400 patients was calculated to allow an 80% power to detect an absolute risk reduction of 10% with an alpha level of 5% in the development of ARDS. The risk of ARDS development was considered in 10% of patients with LIPS score of >4 points. We decided to stop the recruitment 22 months after the inclusion of the first patient. This was due to low recruitment rates and perceived loss of equipoise at all study sites as a comparable randomized clinical trial did not show any benefit of a strategy using a low V_T_ vs. an intermediate V_T_ (7).

### 2.9. Statistical analysis plan

All analyses were conducted as planned, and all analyses followed the intention-to-treat approach.

Categorical variables are reported as numbers and percentages, and quantitative variables are reported as median and interquartile ranges (IQRs). The comparison of V_T_ between groups over time was performed using mixed-effect longitudinal models with random intercepts for hospitals and patients, with day treated as a continuous variable, and with an interaction between days and the randomization group as a fixed effect. The V_T_ in each arm is presented in cumulative distribution plots in the overall population and then stratified according to the use of controlled or assisted ventilation.

For the analysis of the primary endpoint, the risk ratio and 95% confidence intervals (CIs) calculated with the Wald likelihood ratio approximation test and Fisher’s exact tests for hypothesis testing were used. Secondary binary outcomes, i.e., the development of pneumonia or atelectasis, and mortality rates, were also reported as risk ratios with 95% CI determined using the Wald likelihood ratio approximation test and Fisher’s exact tests.

We performed three *post hoc* analyses. First, we compared the number of days free from the ventilator and alive on day 28 between the two groups. This number was calculated by counting the number of calendar days a patient was free from ventilation up to day 28 and giving a penalty of 0 points to patients who died before day 28. The duration of invasive ventilation in survivors was also reported (11). Second, as a sensitivity analysis, we tested the effect of the intervention on outcomes in a mixed-effect (or [shared–frailty] Cox proportional) model with stratification variable (hospital) as random effects and adjusted by LIPS at enrolment. We provide the same numbers of patients in all tables, both in the main text and in the supplement, and we report how many patients we have the data for each variable by showing an (n/N). Of note, we handled the missing data in an adjusted method. Third, we calculated the mechanical power (MP) of ventilation, using the following power equation (12):

MP = 0.098 * RR * V_T_ * [Ppeak – ½ * (Pplat – PEEP)] (in patients under volume-controlled ventilation) and MP = 0.098 * RR * V_T_ * [PEEP + Pinsp] (in patients under pressure-controlled ventilation).

wherein RR is the set respiratory rate; Pplat is the plateau pressure; PEEP is the positive end-expiratory pressure, and ΔPinsp is the difference between maximum airway pressure and PEEP. MP was calculated only for the first 3 days and in patients receiving invasive ventilation with a controlled ventilation mode.

The significance level was set at 0.05, without adjustment for multiple comparisons. Reported *value of p*s are two-sided. All analyses were performed using R software, version 3.6.3 (R Core Team).

## 3. Results

### 3.1. Patients

From 3 November 2014 to 30 August 2016, 98 patients fulfilled the inclusion and exclusion criteria, and all patients were randomized. In total, 50 patients were allocated to the low V_T_ group and 48 patients were allocated to the intermediate V_T_ group. The majority of patients were admitted for medical reasons and intubated because of acute respiratory failure (Table 1).

**Table 1:** Baseline characteristics of the patients.

	Low Tidal Volume (*n* = 50)	Intermediate Tidal Volume (*n* = 48)
Age, years	66 (57–73)	63 (55–71)
Male gender	32/50 (64.0)	34/46 (73.9)
Weight, kilograms	75.0 (65.4–82.5)	80.0 (68.1–85.0)
Body mass index, kg/m^2^	27.2 (23.6–30.6)	26.5 (23.7–31.5)
Predicted body weight, kg	63.8 (57.0–67.8)	64.2 (56.9–67.8)
Prognostic scores
SAPS II score	52 (38–61)	50 (40–61)
APACHE II score	23 (17–26.5)	23 (19–26)
LIPS score	7.5 (6.0–8.9)	6.0 (5.5–7.5)
SOFA score	8 (5–10)	9 (6–11)
Lung injury score	4 (2–6)	4 (3–6)
Septic shock	19/50 (38.0)	18/48 (37.5)
Reason of admission
Medical	29/50 (58.0)	30/46 (65.2)
Emergency surgery	16/50 (32.0)	13/46 (28.3)
Elective surgery	4/50 (8.0)	2/46 (4.3)
Trauma	1/50 (2.0)	1/46 (2.2)
Source of admission
Emergency room	15/50 (30.0)	10/46 (21.7)
Operating room	16/50 (32.0)	13/46 (28.3)
Ward	12/50 (24.0)	15/46 (32.6)
Other ICU	0/50 (0.0)	1/46 (2.2)
Other hospital	0/50 (0.0)	1/46 (2.2)
Others	7/50 (14.0)	6/46 (13.0)
Reason of intubation
Acute respiratory failure	14/48 (29.2)	19/45 (42.2)
Post–surgical	16/48 (33.3)	10/45 (22.2)
Shock	14/48 (29.2)	9/45 (20.0)
Coma	4/48 (8.3)	7/45 (15.6)
Hours ventilated before randomization	7.4 (4.3–10.8)	6.9 (3.7–9.3)
Baseline ventilation variables
Mode of ventilation		
Volume controlled	30/48 (62.5)	30/45 (66.7)
Pressure regulated volume controlled	8/48 (16.7)	11/45 (24.4)
Pressure controlled	8/48 (16.7)	4/45 (8.9)
Pressure support ventilation	2/48 (4.2)	0/45 (0.0)
Tidal volume, mL/kg PBW	8.2 (7.1–9.0)	8.2 (7.6–8.8)
Peak pressure, cmH_2_O	28 (23–32)	26 (22–30)
Plateau pressure, cmH_2_O	18 (15–20)	17 (15–20)
Driving pressure, cmH_2_O	12 (10–14)	12 (9–14)
PEEP, cmH_2_O	5 (5–8)	5 (5–7)
Respiratory rate, bpm	17 (15–23)	17 (15–20)
FiO_2_, %	50 (40–80)	50 (40–70)
Baseline laboratory tests and vital signs
PaO_2_/FiO_2_	250 (171–352)	243 (174–321)
SpO_2_/FiO_2_	196 (119–250)	200 (139–247)
PaCO_2_, mmHg	36.0 (31.6–45.0)	37.2 (31.8–44.0)
pH	7.32 (7.30–7.38)	7.34 (7.25–7.39)
Mean arterial pressure, mmHg	75 (67–87)	70 (64–79)
Heart rate, bpm	89 (76–105)	96 (80–116)

Data are median (quartile 25–quartile 75) or N/Total (%). APACHE, Acute Physiology and Chronic Health Evaluation; FiO2, inspired fraction of oxygen; ICU, intensive care unit; LIPS, lung injury prediction score; PEEP, positive end-expiratory pressure; SAPS, simplified acute physiology score; SOFA, Sequential Organ Failure Assessment.

### 3.2. Intervention

**Figure 1:**
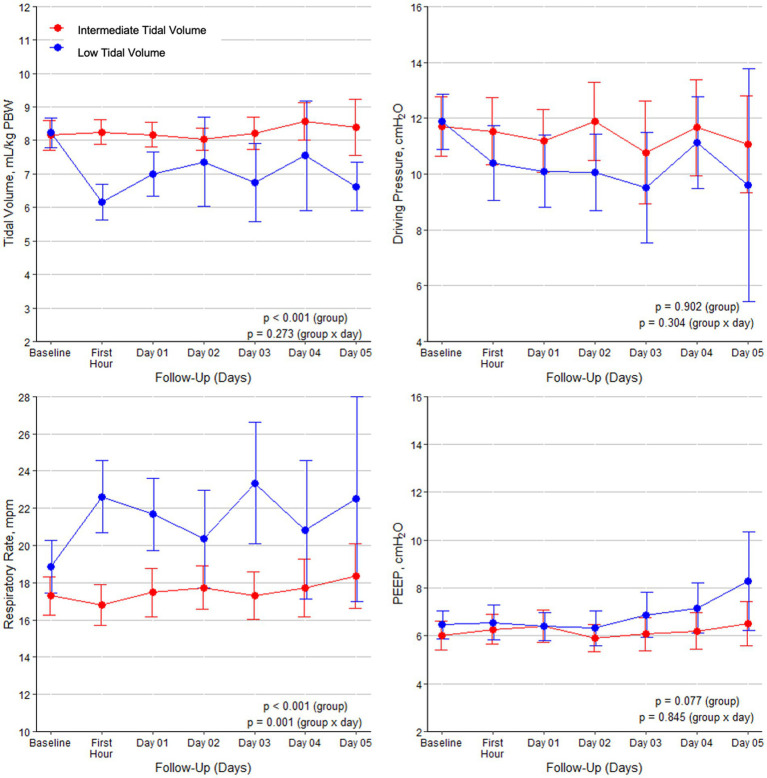
Tidal volumes, driving pressure, respiratory rate, and PEEP during the first 5 days of mechanical ventilation in the two groups. Data are means with 95% confidence intervals.

The median time between the initiation of mechanical ventilation and randomization was 7.1 h (IQR, 4.2–9.9); the median time between the start of ventilation in the ICU and randomization was 0.6 h (IQR, 0.2–1.0). During the initial days of ventilation, the majority of patients in both groups received assisted ventilation (Supplementary Table S1 in the electronic supplement). The interventions led to significantly lower V_T_ in the intervention group (Figure 1). PEEP and driving pressure were not different between the two groups, as were other respiratory variables. MP was not different between the groups (Supplementary Table S1 in the electronic supplement). The incidence of respiratory acidosis was also not different. There were no other clinical differences between the two groups (Supplementary Table S2 in the electronic supplement).

### 3.3. Development of ARDS

On day 7, five (11.9%) patients in the low V_T_ group and four (9.1%) patients in the intermediate V_T_ group had developed ARDS (risk ratio, 1.16 [95% CI, 0.62–2.17]; *p* = 0.735) (Table 2).

**Table 2:** Primary and secondary outcomes.

	Low Tidal Volume (*n* = 50)	Intermediate Tidal Volume (*n* = 48)	Absolute Difference (95% CI)	Effect Estimate (95% CI)	value of *p*
Primary outcome
Development of ARDS within 7 days	5/42 (11.9)	4/44 (9.1)	2.81 (−10.46 to 16.09)^a^	1.16 (0.62 to 2.17)^c^	0.735
Secondary outcomes
Development of pneumonia within 7 days	9/43 (20.9)	9/44 (20.5)	0.48 (−17.00 to 17.95)^a^	1.01 (0.60 to 1.71)^c^	0.999
Development of atelectasis within 7 days	9/43 (20.9)	12/44 (27.3)	−6.34 (−24.75 to 12.06)^a^	0.83 (0.48 to 1.44)^c^	0.617
Ventilator–free days on day 28	24.0 (0.0–27.0)	20.0 (10.2–24.0)	3.68 (0.21 to 7.14)^b^	4.00 (−1.02 to 9.02)^b^	0.122
Duration of ventilation, days	3.0 (1.0–6.0)	7.0 (3.0–14.0)	−4.29 (−6.94 to −1.64)^b^	0.98 (0.55 to 1.75)^e^	0.950
In survivors, days	2.0 (1.0–4.8)	6.5 (3.0–10.0)	−4.67 (−9.62 to 0.29)^b^		
ICU length of stay, days	7.0 (3.8–12.2)	13.5 (6.8–20.5)	−6.07 (−10.01 to −2.13)^b^	1.26 (0.79 to 2.02)^d^	0.327
In survivors, days	8.0 (6.0–13.5)	13.0 (6.5–18.5)	−4.71 (−10.41 to 0.99)^b^		
Hospital length of stay, days	23.5 (13.2–47.2)	29.5 (15.0–42.2)	−5.29 (−17.29 to 6.70)^b^	0.86 (0.53 to 1.38)^d^	0.527
In survivors	31.0 (15.0–58.0)	32.5 (18.0–43.0)	−1.20 (−15.9 to 13.50)^b^		
Mortality
ICU	11/49 (22.4)	8/45 (17.8)	4.67 (−11.95 to 21.29)^a^	1.14 (0.73 to 1.78)^c^	0.615
Hospital	13/46 (28.3)	8/44 (18.2)	10.08 (−7.72 to 27.88)^a^	1.29 (0.85 to 1.96)^c^	0.322
28–day	12/43 (27.9)	8/43 (18.6)	9.30 (−8.92 to 27.52)^a^	2.70 (1.01 to 7.22)^d^	0.047
90–day	12/42 (28.6)	10/43 (23.3)	5.32 (−13.77 to 24.41)^a^	2.63 (0.98 to 7.02)^d^	0.054

Data are median (quartile 25–quartile 75) or N/Total (%). ARDS, acute respiratory distress syndrome; CI, confidence interval; ICU, intensive care unit. ^a^Effect estimate is the risk difference from a generalized linear model with binomial distribution and identity link. ^b^Effect estimate is the median difference from a median regression. ^c^Effect estimate is the risk ratio from a Wald likelihood ratio approximation test and the value of *p* estimated from a Fisher’s exact test. ^d^Effect estimate is the hazard ratio from a Cox proportional hazard model. ^e^Effect estimate is the subdistribution hazard ratio from a Fine-Gray competing risk model with 28-day mortality as a competing risk.

### 3.4. Secondary endpoints

**Figure 2:**
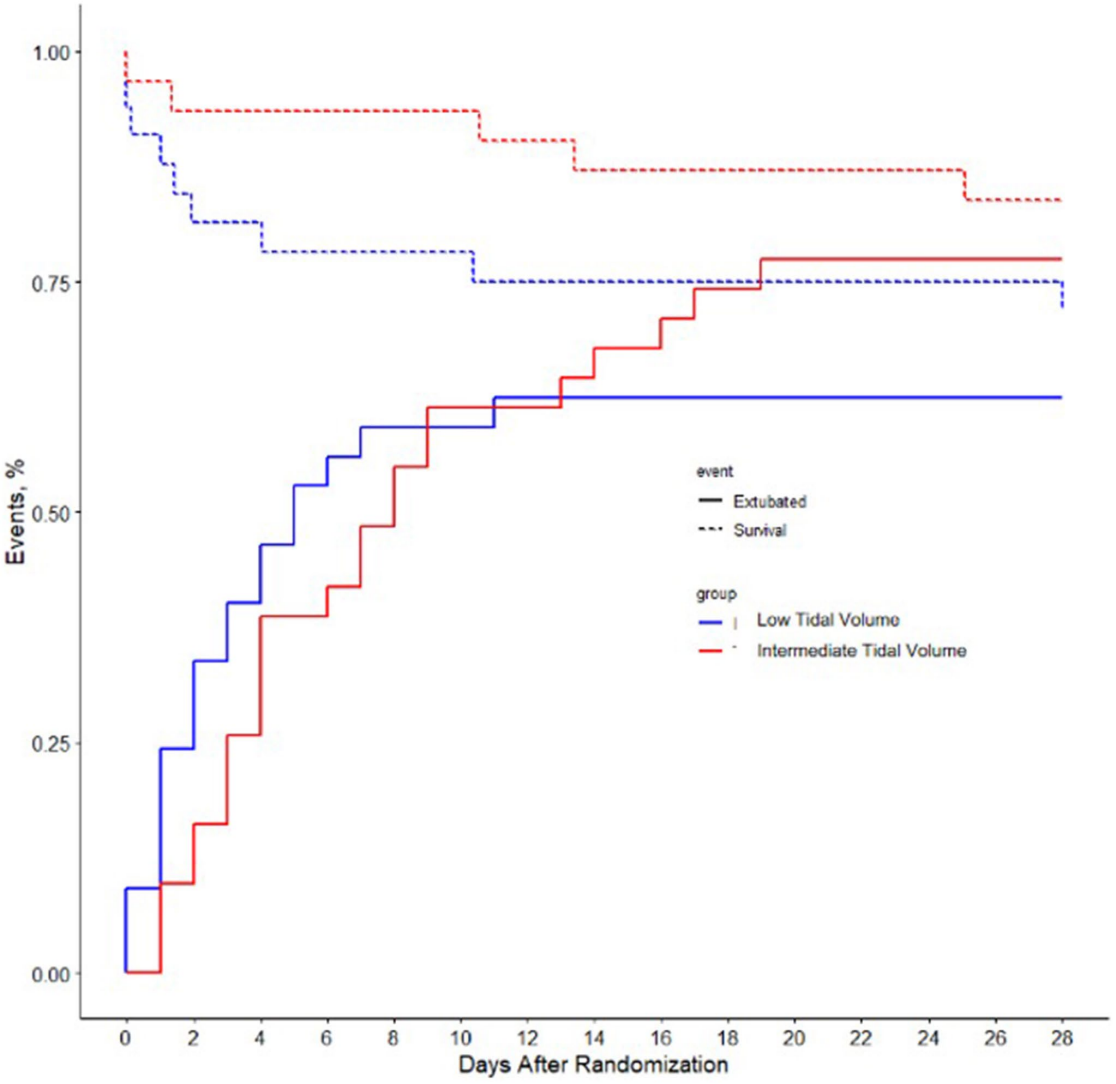
The proportion of patients that experienced successful extubation (solid line) and the proportion of patients that survived till ICU discharge (dashed line). Differences are not statistically significant.

On day 7, the incidence of pneumonia and severe atelectasis was not different between the two groups (Figure 2). There was no difference in ventilator-free days or in the duration of ventilation in survivors. ICU and hospital length of stay were similar, as were in-hospital mortality and 90-day mortality. The mortality at day 28 was lower in the intermediate tidal volume group than in the low tidal volume group.

### 3.5. Sensitivity analysis

The sensitivity analysis did not change the findings, except for 28-day and 90-day mortality that were lower in the intermediate tidal volume group (Supplementary Table S3 in the electronic supplement).

## 4. Discussion

The findings of this randomized clinical trial comparing a low V_T_ strategy with an intermediate V_T_ strategy in patients at risk of ARDS with respect to the development of pulmonary complications can be summarized as follows: (1) the use of a low V_T_ strategy did not change the incidence of ARDS during the first 7 days of ventilation; (2) the use of a low V_T_ strategy also did not affect the development of pneumonia and severe atelectasis; and (3) the use of a low V_T_ strategy was not associated with a shorter duration of ventilation, a higher number of ventilator-free days and alive, a lower length of stay in ICU and hospital, or lower mortality rates.

This study has some strengths. Different from previous studies, we included patients at risk for ARDS and were able to test the hypothesis of whether a low V_T_ strategy would affect the progression to ARDS in such patients. We included consecutive patients with various reasons for invasive ventilation in several ICUs in two countries, which helped in the generalization of our results. Patients were randomized shortly after the start of invasive ventilation in the ICU in order to minimize the effects on the outcome of different ventilation strategies before the start of ventilation according to the protocol. We used a detailed protocol that was strictly followed with similar treatment in both groups except for V_T_ sizes. Finally, we strictly followed a predefined statistical analysis plan, and analyzers were blinded to randomization.

The findings of our study are in line with those from a previous randomized clinical trial, named PReVENT (7). In that study, a ventilation strategy using low V_T_ (4 to 6 mL/kg PBW) was compared to a ventilation strategy using intermediate V_T_ (10 mL/kg PBW) with respect to the number of days free from the ventilator and alive on day 28. The incidence of ARDS in PReVENT (7) was similar to our study and likewise, a low V_T_ strategy was not associated with a lower incidence of ARDS.

Previous studies suggested the benefit of V_T_ reduction in patients not having ARDS (5, 6, 13). Two previous RCTs showed a lower incidence of pneumonia (13) and less development of ARDS (6) in the low V_T_ group. In these studies, a V_T_ of 10 and 12 mL/kg PBW (6, 13) was used in the intermediate arm, which is higher than in our trial where V_T_ in the intermediate arm was lower than 9 mL/kg PBW during the first 5 days of MV. One RCT demonstrated higher inflammation in the intermediate group but did not report any data about lung injury (14).

Two recent individual participant data (IPD) metanalyses (15, 16) suggested that a low VT strategy was associated with a shorter duration of mechanical ventilation (15) and a lower incidence of pulmonary complications (16).

Notably, these IPD analyses showed more difference when low V_T_ was compared with ‘very high’ V_T_ as opposed to ‘high’ or ‘intermediate’ V_T_. This suggests that the difference in outcome in previous RCTs (6, 13) had used V_T_ ≥ 10 mL/kg PBW in the intermediate arm could actually be due to harm from high V_T_ rather than the benefit of low V_T_. However, the IPD meta-analyses included investigations performed over a wide time span, while care, in general, has improved. The difference in results could be due to confounders following changed practices over time including the use of fluids, sedation practices, and the use of neuromuscular blockers.

Our results show that low V_T_ in patients at risk of ARDS does not have a significant impact on reducing the development of ARDS or other pulmonary complications. This is in line with previous results. No adverse effect of using low V_T_ was found. A low V_T_ strategy can lead to a decrease in driving pressure and mechanical power (7), both associated with better outcomes in patients with and without ARDS (17, 18). We believe that some patients could benefit from a low V_T_ strategy, for example, patients with risk factors of lung injury.

The mechanical power of ventilation was not different between the two study groups. Ventilation with a low VT may not reduce MP, since a compensatory increase in respiratory rate could be needed to prevent hypercapnia. This is in line with previous studies (7, 19).

Our study has several limitations. Enrollment was stopped prematurely because of slow recruitment and loss of equipoise caused by publications of similar trials that showed no difference in outcome with regard to pulmonary complications including the development of ARDS (7, 20). This leads to an underpowered sample size, and some data are missing. For this, therefore, conclusions should be drawn with caution. As the trial was stopped for other reasons than those related to the observed intervention effect, this does not predispose it to show disparity in results and should not be considered susceptible to bias due to early discontinuation. There were two important barriers to enrolling patients. First, in contrast to another study that used the deferred informed consent procedure (7), patients or their legal representatives in our study had to provide written informed consent before being included in the study. This was at times challenging, holding in mind that patients needed to be randomized within 12 h after the start of invasive ventilation. The use of deferred informed consent is controversial because it involves enrolling participants in research without their initial informed consent. However, in some cases, it may be the only practical way to conduct research that is important for advancing medical knowledge and improving patient care. The other reason for the low inclusion rate was the use of the LIPS as one of the inclusion criteria. Since this score is not routinely used, it could have delayed the recognition of patients at risk for ARDS. Unfortunately, despite efforts to get complete datasets, we had several outcomes with missing data due to the loss of interest in the study. This, however, happened in both groups. Nevertheless, the trial may have been underpowered to disprove the null hypothesis, and the results need to be interpreted as such. This study included patients in a time window of 5 to 10 years ago. Ventilation practices, however, have hardly changed over the last decade, meaning that the findings of our study still have some general applicability. The reporting of this study was delayed because of several reasons. One reason was the loss of interest in the study question, leading to a decline in the inclusion rate and eventually a complete discontinuation of the study in 2018. The loss of interest also made us decide not to increase the number of participating centers. Then, due to the surges of patients during the COVID-19 pandemic, it was challenging to have the record forms completed and clean and close the database within a reasonable amount of time. The results of this study, however, remain valid and could be used for evidence building regarding the proper size of VT in patients who do not have ARDS, e.g., in systematic reviews and (individual patient data) meta-analyses. Due to the nature of the study, blinding was not possible which is a concern. However, clinicians were unaware of the primary outcome and other endpoints, and there were no differences in care between the groups aside from V_T_.

## 5. Conclusion

In this patient cohort at risk for ARDS, a low V_T_ strategy did not result in a lower incidence of ARDS or other pulmonary complications, compared to an intermediate V_T_ strategy.

## Data availability statement

The raw data supporting the conclusions of this article will be made available by the authors, without undue reservation.

## Ethics statement

The studies involving human participants were reviewed and approved by Institutional Review Board (IRB) of Parc Taulí University Hospital. The patients/participants provided their written informed consent to participate in this study.

## Author contributions

CH is the principal investigator, who conducted the study, interpreted the data, and wrote the manuscript. GG contributed to data collection. AN analyzed and interpreted the patient data. DS contributed to data analysis. MS, FS, and AA were contributors to writing and reviewing the manuscript. MG, AO, CF, FF-V, RG, and FG-V contributed to data collection. All authors contributed to the article and approved the submitted version.

## Conflict of interest

The authors declare that the research was conducted in the absence of any commercial or financial relationships that could be construed as a potential conflict of interest.

## Publisher’s note

All claims expressed in this article are solely those of the authors and do not necessarily represent those of their affiliated organizations, or those of the publisher, the editors and the reviewers. Any product that may be evaluated in this article, or claim that may be made by its manufacturer, is not guaranteed or endorsed by the publisher.

## Abbreviations

ALI: Acute lung injury
APACHE II: Acute Physiology and Chronic Health Classification System
ARDS: Acute respiratory distress syndrome
AUC: Area under the curve
CI: Confidence interval
FiO2: Fraction-inspired oxygen
ICU: Intensive care unit
IQR: Interquartile range
Kg: Kilograms
LIPS: Lung injury prediction score
LIS: Lung injury score
LOS: Length of stay
mL: Milliliters
PaCO2: Arterial pressure carbon dioxide
PaO2: Arterial pressure oxygen
MV: Mechanical ventilation
PBW: Predicted body weight
PEEP: Positive end-expiratory pressure
SOFA: Sequential organ failure assessment
V_T_: Tidal volume
