# Supplementary material for: Effect of a low versus intermediate tidal volume strategy on pulmonary complications in patients at risk of acute respiratory distress syndrome—a randomized clinical trial
Source: Front Med (Lausanne). 2023 Jun 7;10:1172434. doi: 10.3389/fmed.2023.1172434 (PMC10282840; doi:10.3389/fmed.2023.1172434)
Supplement: Supplementary file 1 [file Data_Sheet_1.docx]

Supplementary Material

Effect of a Low vs Intermediate Tidal Volume Strategy on Pulmonary Complications in Patients at risk of ARDS – a randomized clinical trial

Candelaria de Haro ^1,2,^*, Ary Serpa Neto ^3,4,5,6^, Gemma Gomà ^1^, Maria Elena González ^7^,
 Alfonso Ortega ^8^, Catalina Forteza ^9^, Fernando Frutos-Vivar ^10^, Raquel García ^11^, Fabienne D. Simonis ^3^, Federico Gordo–Vidal ^12^, David Suarez ^1^, Marcus J. Schultz ^3,13,14,15,†^ and Antonio Artigas ^1,2,†^

^1^ Critical Care Center, Consorci Corporació Sanitària Parc Taulí, Parc Taulí University Hospital, Institut d’Investigació i Innovació Parc Taulí, Universitat Autònoma de Barcelona, Sabadell, Spain

^2^ CIBER Enfermedades Respiratorias, Instituto de Salud Carlos III, Madrid, Spain

^3^ Department of Intensive Care, Amsterdam University Medical Centers, location ‘AMC’, Amsterdam, The Netherlands

^4^ Department of Critical Care Medicine, Hospital Israelita Albert Einstein, Sao Paulo, Brazil

^5^ Department of Critical Care Medicine, Australian and New Zealand Intensive Care Research Centre (ANZIC-RC), Monash University, Melbourne, Australia

^6^ Department of Critical Care; Data Analytics Research and Evaluation (DARE) Centre, Austin Hospital, Melbourne, Australia

^7^ Intensive Care Unit, Hospital Universitario de Torrejón, Spain

^8^ Intensive Care Unit, Hospital Universitario Puerta de Hierro, Majadahonda, Spain

^9^ Intensive Care Unit, Hospital Son Llàtzer, Palma de Mallorca, Spain

^10^ Intensive Care Unit, Hospital Universitario de Getafe, Spain

^11^ Reanimation Unit, Hospital Universitario 12 de Octubre, Madrid, Spain

^12^ Intensive Care Unit, Hospital del Henares, Grupo de Investigación en Patología Crítica de la Universidad Francisco de Vitoria, Pozuelo de Alarcón, Madrid, Spain

^13^ Department of Medical Affairs, Hamilton Medical AG, Bonaduz, Switzerland

^14^ Mahidol-Oxford Tropical Medicine Research Unit (MORU),Mahidol University, Bangkok, Thailand

^15^ Nuffield Department of Medicine, University of Oxford, Oxford, UK

† These authors contributed equally to this work and share senior authorship.

*** Correspondence:**Corresponding Author
cdeharo@tauli.cat

# Supplementary Data

## e1. Protocol details:

SAFETY MECHANISMS

ARDS development

If ARDS development, tidal volumes in patients in the intermediate-sized tidal volume arm will be decreased to 6mL/kg PBW and they will follow the indication of the ARDSNetwork for ARDS ventilation.

Intolerance criteria

Patients will be evaluated by investigators and removed of the treatment arm, although they will be analyzed in the assigned group, if:

- There is difficult adaptation to mechanical ventilation, evaluated as:
  - - SpO2 < 90%
    - RR > 35bpm
    - Tachycardia > 140
    - SBP < 90mmHg o > 140 mmHg
    - Agitation, sweating, decrease in consciousness level.
- pH < 7.30, despite an increase in respiratory rate until 35rpm and reduction of instrumental death space.

Quality control

To guarantee patients safety, and to guarantee a quality data collection, investigators from enrolling centers will undergo training prior to starting the trial. During the course of the trial, the coordinating center will maintain contact with the enrolling centers with monthly conference calls and periodic auditing of the submitted data. There will be a system to address questions and concerns from enrolling centers.

All data will be collected through an electronic platform, including alarm systems for incorrect data.

All patients will be reviewed and evaluated to detect eventual mistakes in the coordinating center, and queries will be sent. A quality control will be done of 5% of patients (x-ray images, clinical data and analytical data will be asked by the coordinating center) by a second observer.

We will report the progress of the trial 3 times yearly. Any change in the protocol it will be notice to each center, and a new ethical approval will be required.

Adverse events

Adverse events are defined as any undesirable experience occurring to a subject during the study, whether or not considered related to the experimental intervention. All adverse events observed by the investigator or his staff will be recorded (adverse events sheet). Adverse events that are considered related to study procedure include, but are not limited to development of ARDS, development of hypoxemia, hypercarbia, development of pneumothorax.

*Serious adverse events*

A serious adverse event is any untoward medical occurrence or effect that at any circumstance:

- Results in death
- Is life threatening (at the time of the event)
- Requires prolongation of existing inpatients’ hospitalization
- Results in persistent or significant disability or incapacity

Any other important medical event that may not result in death, be life threatening, or requires prolongation of hospitalization, may be considered a serious adverse experience when, based upon appropriate medical judgment, the event may jeopardize the subject or may require an intervention to prevent one of the outcomes listed above.

For this study, SAEs, which according to the principal investigator are related to study procedures, will be directly reported to the DSMB whereas SAEs, which are deemed unrelated to study procedures, will be recorded and reported to the DSMB in a trimestral report.

All AEs will be followed until they have abated, or until a stable situation has been reached. Depending on the event, follow up may require additional tests or medical procedures as indicated, and/or referral to the general physician or a medical specialist. SAEs need to be reported till end of study within, as defined in the protocol.

**eTable 1. Respiratory Variables and Severity During the First Three Days of Treatment.**

|  | | | | | | | | | | | | |
| --- | --- | --- | --- | --- | --- | --- | --- | --- | --- | --- | --- | --- |
|  | **First Hour After Randomization** | | | **Day 1** | | | **Day 2** | | | **Day 3** | | |
|  | **Low**  **Tidal Volume**  **n = 50** | **Intermediate**  **Tidal Volume**  **n = 48** | ***p* value** | **Low Tidal Volume**  **n = 50** | **Intermediate**  **Tidal Volume**  **n = 48** | ***p* value** | **Low**  **Tidal Volume**  **n = 50** | **Intermediate**  **Tidal Volume**  **n = 48** | ***p* value** | **Low**  **Tidal Volume**  **n = 50** | **Intermediate**  **Tidal Volume**  **n = 48** | ***p* value** |
| No of patients | 42/50 (84.0) | 42/48 (87.5) |  | 38 /50(76.0) | 40/48 (83.3) |  | 25/50 (50.0) | 33/48 (68.8) |  | 19/50 (38.0) | 30/48 (62.5) |  |
| Tidal volume, mL/kg PBW | 6.0 (5.7 – 6.4) | 8.4 (8.0 – 8.8) | < 0.001 | 6.2 (5.9 – 7.8) | 8.1 (7.9 – 8.9) | < 0.001 | 7.1 (5.8 – 8.1) | 8.0 (7.7 – 8.5) | 0.035 | 6.7 (5.6 – 7.2) | 8.1 (7.7 – 8.8) | 0.002 |
| ≤ 6 mL/kg PBW | 15 (57.7) | 1 (3.2) | < 0.001 | 10 (30.3) | 2 (5.7) | 0.019 | 5 (33.3) | 1 (3.7) | 0.030 | 5 (41.7) | 1 (4.2) | 0.018 |
| 8–10 mL/kg PBW | 1 (3.8) | 24 (77.4) | < 0.001 | 5 (15.2) | 19 (54.3) | 0.002 | 4 (26.7) | 13 (48.1) | 0.303 | 1 (8.3) | 11 (45.8) | 0.061 |
| > 10 mL/kg PBW | 1 (3.8) | 0 (0.0) | 0.929 | 3 (9.1) | 1 (2.9) | 0.564 | 1 (6.7) | 0 (0.0) | 0.763 | 1 (8.3) | 1 (4.2) | 1.000 |
| Peak pressure, cmH_2_O | 23 (18 – 31) | 26 (21 – 30) | 0.284 | 24 (19 – 30) | 26 (21 – 32) | 0.267 | 23 (17 – 30) | 26 (21 – 32) | 0.319 | 22 (16 – 25) | 23 (19 – 30) | 0.212 |
| Plateau pressure, cmH_2_O | 18 (15 – 20) | 17 (15 – 20) | 0.944 | 17 (14 – 19) | 17 (15 – 20) | 0.408 | 17 (15 – 19) | 17 (15 – 21) | 0.480 | 18 (15 – 18) | 16 (14 – 20) | 0.919 |
| Driving pressure, cmH_2_O | 11 (9 – 12) | 11 (9 – 13) | 0.466 | 10 (8 – 11) | 11 (9 – 13) | 0.159 | 9 (9 – 11) | 11 (10 – 15) | 0.076 | 10 (8 – 12) | 10 (8 – 15) | 0.347 |
| PEEP, cmH_2_O | 5 (5 – 8) | 5 (5 – 8) | 0.779 | 6 (5 – 7) | 5 (5 – 8) | 0.698 | 5 (5 – 8) | 5 (5 – 6) | 0.271 | 7 (5 – 8) | 5 (5 – 7) | 0.082 |
| Respiratory rate, mpm  Mechanical power (J/min)* | 22 (18 – 26)  20.51 (14.90 – 27.59) | 6 (14 – 20)  17.58 (12.54 – 22.56) | < 0.001  0.059 | 22 (18 – 26)  19.46 (13.53 – 22.94) | 16 (15 – 20)  19.04 (12.85 – 23.92) | 0.002  0.705 | 20 (15 – 24)  19.83 (14.92 – 21.20) | 18 (16 – 20)  18.65 (10.84 – 23.64) | 0.148  0.910 | 22 (18 – 26)  18.60 (13.68 – 22.34) | 16 (15 – 20)  16.46 (9.24 – 24.91) | <0.001  1.000 |
| FiO_2_, % | 40 (35 – 50) | 40 (40 – 50) | 0.886 | 40 (35 – 50) | 40 (35 – 40) | 0.627 | 37 (30 – 40) | 40 (35 – 40) | 0.922 | 35 (30 – 41) | 35 (31 – 40) | 0.811 |
| PaO_2_ / FiO_2_ | 282 (212 – 331) | 249 (200 – 336) | 0.544 | 303 (214 – 349) | 265 (229 – 322) | 0.602 | 306 (274 – 340) | 260 (223 – 337) | 0.100 | 310 (226 – 394) | 257 (222 – 314) | 0.292 |
| SpO_2_ / FiO_2_ | 241 (193 – 274) | 245 (196 – 250) | 0.963 | 250 (200 – 286) | 250 (241 – 277) | 0.671 | 262 (246 – 313) | 250 (245 – 286) | 0.816 | 267 (232 – 318) | 267 (246 – 306) | 0.862 |
| PaCO_2_, mmHg | 37.1 (32.6 – 43.0) | 36.0 (33.5 – 40.9) | 0.552 | 38.5 (33.8 – 43.3) | 35.5 (32.1 – 39.1) | 0.065 | 40.1 (38.0 – 43.0) | 36.8 (34.0 – 42.0) | 0.065 | 40.6 (37.5 – 43.8) | 37.5 (33.4 – 40.0) | 0.088 |
| pH | 7.33 (7.28 – 7.37) | 7.36 (7.31 – 7.39) | 0.110 | 7.37 (7.30 – 7.39) | 7.39 (7.33 – 7.44) | 0.036 | 7.35 (7.32 – 7.39) | 7.39 (7.36 – 7.44) | 0.036 | 7.39 (7.36 – 7.42) | 7.42 (7.39 – 7.45) | 0.067 |
| pH < 7.25 | 5 (12.8) | 7 (17.9) | 0.754 | 3 (8.3) | 2 (5.0) | 0.903 | 2 (9.5) | 1 (3.0) | 0.685 | 0 (0.0) | 0 (0.0) | --- |
| Respiratory acidosis | 5 (12.8) | 4 (10.3) | 1.000 | 2 (5.6) | 1 (2.5) | 0.926 | 2 (9.5) | 0 (0.0) | 0.286 | 3 (18.8) | 2 (6.7) | 0.449 |
| Severity |  |  |  |  |  |  |  |  |  |  |  |  |
| Total SOFA | --- | --- | --- | 7 (5 – 10) | 8 (6 – 11) | 0.224 | --- | --- | --- | 4 (3 – 8) | 7 (6 – 10) | 0.012 |
| Lung injury score | --- | --- | --- | 3 (2 – 5) | 3 (2 – 5) | 0.862 | --- | --- | --- | 2 (2 – 5) | 4 (3 – 6) | 0.031 |
| Use of NMBA | --- | --- | --- | 1 (2.6) | 1 (2.6) | 0.999 | 1 (2.9) | 0 (0.0) | 0.999 | 0 (0.0) | 0 (0.0) | --- |
| Data are median (quartile 25 – quartile 75) or N / Total (%)  *No of patients: First hour after randomization (43/50 Low Tidal Volume vs 41/48 Intermediate Tidal Volume); Day 1 (26/50 Low Tidal Volume vs 36/48 Intermediate Tidal Volume); Day 2 (14/50 Low Tidal Volume vs 30/48 Intermediate Tidal Volume); Day 3 (9/50 Low Tidal Volume vs 23/48 Intermediate Tidal Volume);  *FiO2: inspired fraction of oxygen; NMBA: neuromuscular blocking agents; PBW: predicted body weight; PEEP: positive-end expiratory pressure; SOFA: Sequential Organ Failure Assessment* | | | | | | | | | | | | |

| **eTable 2 – Physiological Variables, Laboratory Tests, Co–interventions and Complications During the First Three Days** | | | | | | | | | |
| --- | --- | --- | --- | --- | --- | --- | --- | --- | --- |
|  | **Day 1** | | | **Day 2** | | | **Day 3** | | |
|  | **Low**  **Tidal Volume**  **n = 50** | **Intermediate**  **Tidal Volume**  **n = 48** | ***p* value** | **Low**  **Tidal Volume**  **n = 50** | **Intermediate**  **Tidal Volume**  **n = 48** | ***p* value** | **Low**  **Tidal Volume**  **n = 50** | **Intermediate**  **Tidal Volume**  **n = 48** | ***p* value** |
| No of patients | 38/50 (76.0) | 40 /48(83.3) |  | 25/50 (50.0) | 33/48 (68.8) |  | 19/50 (38.0) | 30/48 (62.5) |  |
| Physiological variables |  |  |  |  |  |  |  |  |  |
| Heart rate, bpm | 80 (68 – 99) | 91 (79 – 107) | 0.046 | 80 (71 – 98) | 82 (75 – 99) | 0.585 | 75 (68 – 85) | 79 (72 – 88) | 0.369 |
| Mean arterial pressure, mmHg | 73 (65 – 84) | 80 (67 – 88) | 0.227 | 80 (70 – 85) | 82 (74 – 89) | 0.279 | 84 (75 – 90) | 82 (74 – 87) | 0.565 |
| Fluid balance, mL | 2090 (392 – 3710) | 1951 (217 – 3370) | 0.992 | 1004 (-123 – 2216) | 696 (-46 – 2205) | 0.999 | 0 (-1280 – 1145) | 162 (-781 – 1559) | 0.264 |
| Laboratory tests |  |  |  |  |  |  |  |  |  |
| Hemoglobin, g/dL | 10.4 (8.8 – 12.0) | 10.1 (9.1 – 11.1) | 0.384 | 9.6 (8.1 – 10.9) | 9.7 (8.6 – 10.5) | 0.670 | 9.4 (8.7 – 10.7) | 9.1 (8.5 – 10.0) | 0.586 |
| Lactate, mg/dL | 13.5 (9.5 – 18.3) | 17.0 (8.9 – 30.5) | 0.715 | 10.8 (8.0 – 16.4) | 14.1 (9.9 – 23.0) | 0.185 | 12.2 (7.2 – 15.5) | 12.6 (8.1 – 17.1) | 0.713 |
| White blood cell count, cels x 1000/mm^3^ | 14.3 (8.8 – 22.0) | 12.5 (7.6 – 18.2) | 0.368 | 11.8 (9.1 – 18.6) | 11.2 (7.1 – 22.5) | 0.719 | 9.1 (6.4 – 12.9) | 9.8 (6.9 – 19.4) | 0.374 |
| C–reactive protein (mg/dL) | 19.9 (8.3 – 37.5) | 15.1 (8.5 – 31.4) | 0.375 | 23.6 (12.5 – 37.8) | 16.9 (8.8 – 31.0) | 0.144 | 14.2 (7.8 – 21.0) | 13.8 (7.8 – 22.5) | 0.970 |
| Pro–calcitonin (ng/mL) | 4.9 (1.1 – 15.1) | 10.8 (0.8 – 35.2) | 0.566 | 2.5 (1.4 – 7.6) | 11.7 (6.4 – 18.8) | 0.110 | 1.4 (1.0 – 2.3) | 6.1 (2.1 – 8.5) | 0.129 |
| Creatinine, mg/dL | 1.42 (0.80 – 1.97) | 1.71 (0.83 – 2.42) | 0.213 | 0.95 (0.63 – 1.56) | 1.08 (0.72 – 1.89) | 0.415 | 0.90 (0.60 – 1.35) | 0.80 (0.68 – 1.80) | 0.604 |
| Bilirubin, mg/dL | 0.5 (0.3 – 1.0) | 0.9 (0.5 – 4.2) | 0.013 | 0.4 (0.3 – 0.6) | 0.9 (0.3 – 2.5) | 0.014 | 0.4 (0.3 – 0.5) | 0.8 (0.3 – 2.6) | 0.021 |
| Adjuvant therapies |  |  |  |  |  |  |  |  |  |
| Use of corticosteroids | 16 (42.1) | 17 (43.6) | 0.999 | 17 (48.6) | 14 (41.2) | 0.707 | 11 (39.3) | 12 (35.3) | 0.952 |
| Use of norepinephrine | 29 (76.3) | 31 (79.5) | 0.952 | 24 (68.6) | 24 (70.6) | 0.999 | 16 (57.1) | 17 (50.0) | 0.760 |
| Use of dobutamine | 12 (31.6) | 11 (28.2) | 0.941 | 7 (20.0) | 10 (29.4) | 0.530 | 9 (32.1) | 12 (35.3) | 0.999 |
| Use of dopamine | 9 (23.7) | 8 (20.5) | 0.952 | 6 (17.1) | 7 (20.6) | 0.954 | 9 (32.1) | 9 (26.5) | 0.835 |
| Need of renal replacement therapy | 8 (22.2) | 10 (26.3) | 0.889 | 5 (14.7) | 7 (20.6) | 0.750 | 5 (17.9) | 8 (24.2) | 0.769 |
| Complications |  |  |  |  |  |  |  |  |  |
| Acute kidney injury | 19 (52.8) | 28 (73.7) | 0.104 | 15 (44.1) | 23 (67.6) | 0.087 | 9 (32.1) | 20 (60.6) | 0.050 |
| Sepsis | 25 (69.4) | 23 (62.2) | 0.683 | 21 (61.8) | 18 (52.9) | 0.624 | 12 (42.9) | 15 (45.5) | 0.999 |
| Data are median (quartile 25 – quartile 75) or N / Total (%) | | | | | | | | | |

**eTable 3. Primary and Secondary Outcomes in the Sensitivity Analysis**

|  | | | | |
| --- | --- | --- | --- | --- |
|  | **Low**  **Tidal Volume**  **(*n* = 50)** | **Intermediate**  **Tidal Volume**  **(*n* = 48)** | **Adjusted Effect Estimate***  **(95% CI)** | ***p* value** |
| **Primary outcome** |  |  |  |  |
| Development of ARDS within 7 days | 5 / 42 (11.9) | 4 / 44 (9.1) | 1.35 (0.33 to 5.54)^a^ | 0.676 |
| **Secondary outcomes** |  |  |  |  |
| Development of pneumonia within 7 days | 9 / 43 (20.9) | 9 / 44 (20.5) | 0.77 (0.23 to 2.59)^a^ | 0.674 |
| Development of atelectasis within 7 days | 9 / 43 (20.9) | 12 / 44 (27.3) | 0.86 (0.22 to 3.36)^a^ | 0.830 |
| Ventilator–free days at day 28 | 24.0 (0.0 - 27.0) | 20.0 (10.2 - 24.0) | 4.00 (0.30 to 7.69)^b^ | 0.036 |
| Duration of ventilation, days | 3.0 (1.0 - 6.0) | 7.0 (3.0 - 14.0) | 1.16 (0.72 to 1.86)^d^ | 0.550 |
| In survivors, days | 2.0 (1.0 - 4.8) | 6.5 (3.0 - 10.0) |  |  |
| ICU length of stay, days | 7.0 (3.8 - 12.2) | 13.5 (6.8 - 20.5) | 1.40 (0.85 to 2.32)^c^ | 0.190 |
| In survivors, days | 8.0 (6.0 - 13.5) | 13.0 (6.5 - 18.5) |  |  |
| Hospital length of stay, days | 23.5 (13.2 - 47.2) | 29.5 (15.0 - 42.2) | 0.89 (0.54 to 1.47)^c^ | 0.660 |
| In survivors | 31.0 (15.0 - 58.0) | 32.5 (18.0 - 43.0) |  |  |
| Mortality |  |  |  |  |
| ICU | 11 / 49 (22.4) | 8 / 45 (17.8) | 1.21 (0.43 to 3.43)^a^ | 0.710 |
| Hospital | 13 / 46 (28.3) | 8 / 44 (18.2) | 1.56 (0.55 to 4.39)^a^ | 0.400 |
| 28–day | 12 / 43 (27.9) | 8 / 43 (18.6) | 3.72 (1.30 to 10.66)^c^ | 0.014 |
| 90–day | 12 / 42 (28.6) | 10 / 43 (23.3) | 3.49 (1.22 to 9.94)^c^ | 0.019 |
| Data are median (quartile 25 – quartile 75) or N / Total (%)  *ARDS: acute respiratory distress syndrome; CI: confidence interval; ICU: intensive care unit*  * with stratification variable (hospital) as random effects and adjusted by LIPS score at enrolment  ^a^ effect estimate is odds ratio from a mixed–effect generalized linear model with binomial distribution  ^b^ effect estimate is median difference from a mixed–effect median regression  ^c^ effect estimate is hazard ratio from a (shared–frailty) Cox proportional hazard model  ^d^ effect estimate is subdistribution hazard ratio from a Fine-Gray competing risk model with 28–day mortality as competing risk and with centres as cluster | | | | |
